# A Phenomenological Model Reproducing Temporal Response Characteristics of an Electrically Stimulated Auditory Nerve Fiber

**DOI:** 10.1177/23312165221117079

**Published:** 2022-09-07

**Authors:** Marko Takanen, Bernhard U. Seeber

**Affiliations:** 1 Audio Information Processing, Department of Electrical and Computer Engineering, Technical University of Munich, Munich, Germany

**Keywords:** auditory model, electrical stimulation, cochlear implant, inter-pulse interaction, single-fiber recordings

## Abstract

The ability of cochlear implants (CIs) to restore hearing to profoundly deaf people is based on direct electrical stimulation of the auditory nerve fibers (ANFs). Still, CI users do not achieve as good hearing outcomes as their normal-hearing peers. The development and optimization of CI stimulation strategies to reduce that gap could benefit from computational models that can predict responses evoked by different stimulation patterns, particularly temporal responses for coding of temporal fine structure information. To that end, we present the sequential biphasic leaky integrate-and-fire (S-BLIF) model for the ANF response to various pulse shapes and temporal sequences. The phenomenological S-BLIF model is adapted from the earlier BLIF model that can reproduce neurophysiological single-fiber cat ANF data from single-pulse stimulations. It was extended with elements that simulate refractoriness, facilitation, accommodation and long-term adaptation by affecting the threshold value of the model momentarily after supra- and subthreshold stimulation. Evaluation of the model demonstrated that it can reproduce neurophysiological data from single neuron recordings involving temporal phenomena related to inter-pulse interactions. Specifically, data for refractoriness, facilitation, accommodation and spike-rate adaptation can be reproduced. In addition, the model can account for effects of pulse rate on the synchrony between the pulsatile input and the spike-train output. Consequently, the model offers a versatile tool for testing new coding strategies for, e.g., temporal fine structure using pseudo-monophasic pulses, and for estimating the status of the electrode-neuron interface in the CI user's cochlea.

## Introduction

Receiving a cochlear implant (CI) provides a substantial improvement in the quality of life of most profoundly deaf patients by restoring their hearing and allowing them to understand speech again. To that end, the external speech-processor unit must encode the captured acoustical signal into trains of electrical pulses that are then emitted from the electrodes in the array implanted inside the cochlea to stimulate the auditory nerve fibers (ANFs) directly (for a review, see, e.g., Loizou, 1998) – bypassing the mechano-electrical transduction. Ideally, such an electrical stimulation would result in similar spiking information traversing to the higher stages of the auditory system in comparison to that what is conveyed when the ANFs are able to receive synaptic input from the inner hair cells in a healthy ear. Channel interaction (Stickney et al., 2006; White et al., 2000) and dynamic-range (Zeng, 2004) related limitations of the electrode-nerve interface set profound challenges for achieving that goal. In most CI sound-coding strategies, amplitude-modulated, fixed-rate pulse trains are emitted from the electrodes in an interleaved manner, and the current levels of the pulses are adjusted according to the envelopes extracted from the captured acoustical signal in fixed frequency regions (Wilson et al., 1991). Due to the fixed pulse rate, the temporal fine structure of the acoustical signal is not conveyed in the electrical pulse trains. The lack of conveyed temporal fine structure may at least partially explain why bilateral CI users continue to face severe challenges in localizing sounds and understanding speech in complex, everyday-life listening environments where other sound sources and reverberation hinder the performance of the listener (see, e.g., Badajoz-Davila et al., 2020; Friesen et al., 2001; Kerber & Seeber, 2012, 2013; Zheng et al., 2011).

The challenges faced by CI users continue to motivate researchers and device manufacturers to develop better sound-coding strategies. To that end, there is an evident need to know how the ANF responds to a particular stimulation, and that is known to depend on several characteristics of the stimulus and the ANF itself. Such information can be obtained only via neurophysiological single-fiber measurements on other mammals or by analyzing compound neural responses from CI users. The use of computational models for predicting the responses evoked by a given stimulation provides an interesting alternative for such time-consuming approaches for optimizing coding strategies. Moreover, phenomenological models offer an additional benefit in comparison to biophysical models by being more easily tuned to individual CI users due to their limited parameter space.

One possible method for improving temporal coding in electrical stimulation could be achieved by using novel pulse shapes that are still charge-balanced, like the currently used symmetric biphasic pulses, but could offer a more predictable response timing due to their asymmetric and/or non-rectangular shape (Ballestero et al., 2015; Shepherd & Javel, 1999). Horne et al. (2016) presented a phenomenological model that can accurately reproduce physiological data from single-pulse stimulation with various (monophasic or symmetric/asymmetric charge-balanced biphasic) pulse shapes. Together with the point-process model by Goldwyn et al. (2012), the BILF model of Horne et al. (2016) was one of the first phenomenological models to consider both polarities in charge-balanced pulses. It distinguished itself from earlier phenomenological models (for a review, see Takanen et al., 2016) by being able to reproduce the effects of phase- and inter-phase-gap durations on spiking probability and from thereon to the time of spiking, considering spike latency and temporal jitter. However, for a pulse-train stimulus, it would predict only whether the neuron would spike after its threshold value is exceeded for the first time and, if so, output the predicted time of spiking.

Here, we present a further developed BLIF model, called the sequential biphasic leaky integrate-and-fire (S-BLIF) model, that has been extended for pulse-train stimulation, where several temporal phenomena related to inter-pulse interactions affect the responsiveness of the ANF during the stimulation (Boulet et al., 2016). To that end, we have added elements that simulate the refractory and short- and long-term recovery behavior of the ANF by increasing the threshold value of the modeled neuron temporarily upon spiking. On the other hand, another added element reduces the threshold value temporarily after sub-threshold stimulation in order to emulate the active component of facilitation (Hodgkin, 1938). The fundamental principle guiding the development process was to achieve a versatile model of as low complexity and limited parameter space as possible so that the model could be conveniently tuned for individual CI users. The evaluations presented below demonstrate that the revised model can reproduce neurophysiological data from the literature involving refractoriness, facilitation, accommodation, and spike-rate adaptation phenomena that all affect the responsiveness of the ANF to individual stimulation pulses and how well the timings of the pulses are represented in the spike timings of the neuron. It should be noted that similar aspects or a subset of them have been qualitatively reproduced also by existing phenomenological models (Bruce et al., 1999; Bruce et al., 2000; Campbell et al., 2012; Fredelake & Hohmann, 2012; Goldwyn et al., 2012; Hamacher, 2003; Joshi et al., 2017; Van Gendt et al., 2016). There are also biophysical ANF models that have been able to account for temporal phenomena in pulsatile stimulation (Boulet & Bruce, 2017; Negm & Bruce, 2014). Here, however, a simple single-integrator model is shown to be able to reproduce the aforementioned data quantitatively, whereas previous phenomenological models have either needed two separate integrators to consider both polarities in charge-balanced pulses (Goldwyn et al., 2012; Joshi et al., 2017) or made no distinction between monophasic, pseudo-monophasic or biphasic pulses for the sake of simplicity (Bruce et al., 1999; 2000; Fredelake & Hohmann, 2012; Hamacher, 2003; Van Gendt et al., 2016).

## Methods

The S-BLIF model is implemented in MATLAB (Mathworks, Natick, MA) and it is publicly available at DOI:10.5281/zenodo.4674563. The present model builds on the phenomenological biphasic leaky integrate-and-fire (BLIF) model by Horne et al. (2016), extending it for pulse-train stimulation. Therefore, the functionality of the BLIF model is first briefly reviewed before describing the structure and functionality of the present model.

### BLIF Model

Following the traditional leaky integrate-and fire (LIF) modeling principle (Lapicque, 1907), the BLIF model by Horne et al. (2016) uses a first-order low-pass filter to emulate how the electrical stimulation charges up the ANF's capacitive membrane potential V(t) while some of the charge is lost due to the membrane leakage resistance. Time-constant τ of the low-pass filter is set at 248 µs, which allows the BLIF model to account for the dependency of the threshold value on the pulse duration (Van Den Honert & Stypulkowski, 1984). For simplicity, the neuron's resting potential is set at 0 V and the time-varying threshold Θ(t) is assumed to follow a normal distribution $N(\mu_{\mathrm{THR}},\sigma_{\mathrm{THR}}^{2})$ where the variance $\sigma_{\mathrm{THR}}^{2}$ is related to channel noise (White et al., 2000). The threshold-crossing detector in the BLIF model searches for the time instant $t_{0}$ at which V(t) first exceeds the stochastic threshold value Θ(t) . It should be noted that other models (e.g., Joshi et al., 2017; Tabibi et al., 2021) are using 1/f noise to more closely mimic fluctuations in cell-membrane potentials. A white Gaussian noise distribution was used by Horne et al. (2016) as it yielded comparable results for clinically relevant pulse durations, is easier to generate and enabled using the same distribution for simulating the dependency of the jitter and latency on the spiking probability, as explained below.

In a traditional LIF model, such a threshold-crossing detector would generate an *ad hoc* action potential immediately upon the membrane potential reaching the neuron's threshold value. However, neurophysiological experiments with charge-balanced biphasic pulses, used in current CIs, have revealed that the lagging phase of the biphasic pulse can cancel out the action potential that the leading excitatory phase would have otherwise evoked (Van Den Honert & Mortimer, 1979; Weitz et al., 2011). In addition, and in relation to the above, the threshold value is generally higher for biphasic pulses than for equivalent monophasic pulses (Shepherd & Javel, 1999). To account for these phenomena, the action-potential process is divided in the BLIF model into two events – an initiation process that starts immediately at $t_{0}$ , and computation of the time of spiking that starts only upon successful completion of the initiation process. Completion of the initiation process is designed to occur after an exponentially distributed duration $t_{1}$ , with a minimum duration of 35 µs and the expected value depending on the model's prediction for the response jitter (Horne et al., 2016). The estimated time-of-completion of the initiation process is then compared against the time point $T_{Q0}\;$ at which the lagging phase increases the integrated current above the value at t0, i.e., below the neuron's stochastic threshold for this pulse. In other words, if $t_{1}>\;T_{Q0}$ , the action-potential generation process is terminated and the otherwise to-be-generated spike is canceled. This can be interpreted as hyperpolarization of the neuron which occurs at the next zero crossing of the cumulative charge, computed starting from the threshold crossing to the first pulse phase. The implementation of the S-BLIF model presented here differs from the original implementation by Horne et al. (2016) in that it equally functions with cathodic-first and anodic-first biphasic pulses since the integrated current is compared to a negative and a positive threshold and the subsequent zero-crossing finding is polarity independent. This reflects the observation that the ANF's threshold is largely independent of the polarity of the first phase (Macherey et al., 2006).

The actual time of spiking $t_{\mathrm{spk}}$ is then estimated in the last stage of the BLIF model by introducing a stochastic delay after the threshold crossing

(1) $$\begin{array}{c} t_{\mathrm{spk}}=t_{0}+\Delta (t)∼N(\mu_{\mathrm{latency}},\sigma_{\mathrm{jitter}}^{2}) \end{array}$$

The purpose of this step is to account for the stochastic delay between the onset of the electrical stimulus and the time when the action potential released by a real neuron can be observed in the recording electrode (Miller et al., 1999). Furthermore, the average response delay, called latency, and the standard deviation of the delay, called jitter, are both known to decrease with increased stimulation level (Mino et al., 2002; Rubinstein, 1995). To emulate such dependency, the values for $\mu_{\mathrm{latency}}\;$ and $\sigma_{\mathrm{jitter}}^{2}$ are modeled in the BLIF model as functions of the continuously estimated probability of spiking upon threshold crossing (Horne et al., 2016).

### Sequential Biphasic Leaky Integrate-and-Fire Model (S-BLIF)

The S-BLIF model is designed to represent one (either the peripheral or the central) site of excitation of the auditory nerve fiber that can be excited by both anodic- and cathodic-leading (monophasic, biphasic or triphasic) pulses with the charge-balancing polarity (in bi- or triphasic pulses) being capable of canceling the spiking as described above (Horne et al., 2016). Specifically, the charge delivered by the pulse(s) builds up the membrane voltage (in either the positive or the negative direction) and the modeled neuron then spikes if the membrane voltage exceeds – in absolute terms – either the threshold for anodic or cathodic pulses, unless the spiking activity is abolished by the charge-balancing polarity before the modeled neuron is ready to spike. Table 1 summarizes the parameters of the S-BLIF model. The negative (for anodic polarity) and positive (for cathodic polarity) threshold values could be fitted and interpreted analogous to how distal and proximal parts of an ANF are excited by the anodic and cathodic polarities, respectively. Equal threshold and latency values are used here for both polarities in light of contradictory findings from neurophysiological recordings with monophasic pulses: In cat ANFs, an anodic pulse has been found to require higher stimulation levels but to result in shorter latencies (Miller et al., 1999), being thus more likely exciting the central part of the neuron. In contrast, the opposite difference in threshold values is observed in guinea pigs (Miller et al., 1998). Human CI users have also been found to be more sensitive to charge-balanced biphasic pulses when the leading, excitatory polarity is anodic (Macherey et al., 2008; Undurraga et al., 2010). To further complicate the matter, other studies have found equal threshold values for both polarities (Macherey et al., 2006). In fact, it has been suggested that the found polarity differences in single-fiber recordings may be related to the cochlear location of the stimulating electrode (Ranck, 1975). In accordance with the selected model principle, another likely explanation for the dependency of the sensitivity around the stimulating electrode is the degree of myelinization and degeneration of peripheral parts of the nuclei near the electrode (McIntyre & Grill, 2000; Rattay et al., 2001; Zhou et al., 1995).

**Table 1.** Parameters of the Sequential Biphasic Leaky Integrate-and-Fire (S-BLIF) Model as Applied in all Simulations of the Present Study. the List Contains Only Parameters That Were Introduced When Extending the BLIF Model (Horne et al., 2016) for Pulse-Train Stimulation, Excluding all Parameters of the BLIF Model as They Were not Modified in the Process. Full List of Parameters of the Original BLIF Model Can Be Found in Horne et al. (2016).

Parameter	Description	Value
$\theta_{C}$	Threshold value for cathodic polarity	104.54 µV
$\theta_{A}$	Threshold value for anodic polarity	−104.54 µV
q	Constant in modeling refractoriness	0.76
r	Constant in modeling refractoriness	8.77 × 10^−3^
$t_{A\mathrm{RP}}$	Parameter for absolute refractory time	0.3 ms
$\tau_{\mathrm{RRP}}$	Time constant for relative refractory period	$\tau_{\mathrm{RRP}}∼N(1.5,\;0.4)$ ms
$c_{a}$	Coefficient for increasing the threshold value in modeling long-term adaptation	$c_{a}∼N(0.01,\;0.01)$
$t_{a}$	Time constant for long-term adaptation	125 ms
$m_{a}$	Maximum increase of threshold by long-term adaptation	1.38
$a_{0}$	Offset term in modeling active component of facilitation	1.30 × 10^−9^
$a_{1}$	First-order polynomial coefficient in modeling active component of facilitation	−2.42 × 10^−6^
$a_{2}$	Second-order polynomial coefficient in modeling active component of facilitation	1.68 × 10^−3^
$a_{3}$	Third-order polynomial coefficient in modeling active component of facilitation	0.51

Hence, it was deemed practical to not introduce differences in absolute threshold values between cathodic and anodic pulses *per se* but to construct a model where differences in threshold and latency characteristics are possible. Despite this simplification, the approach is versatile as models with different parameter values can be combined to consider both peripheral and central site of excitation (Takanen & Seeber, 2016; Werner & Seeber, 2018) and/or the parameters of the modeled neurons can be varied in a population model (Werner et al., 2018). In the simulations presented below, the model was always used with the same parameter values.

**Figure 1.**
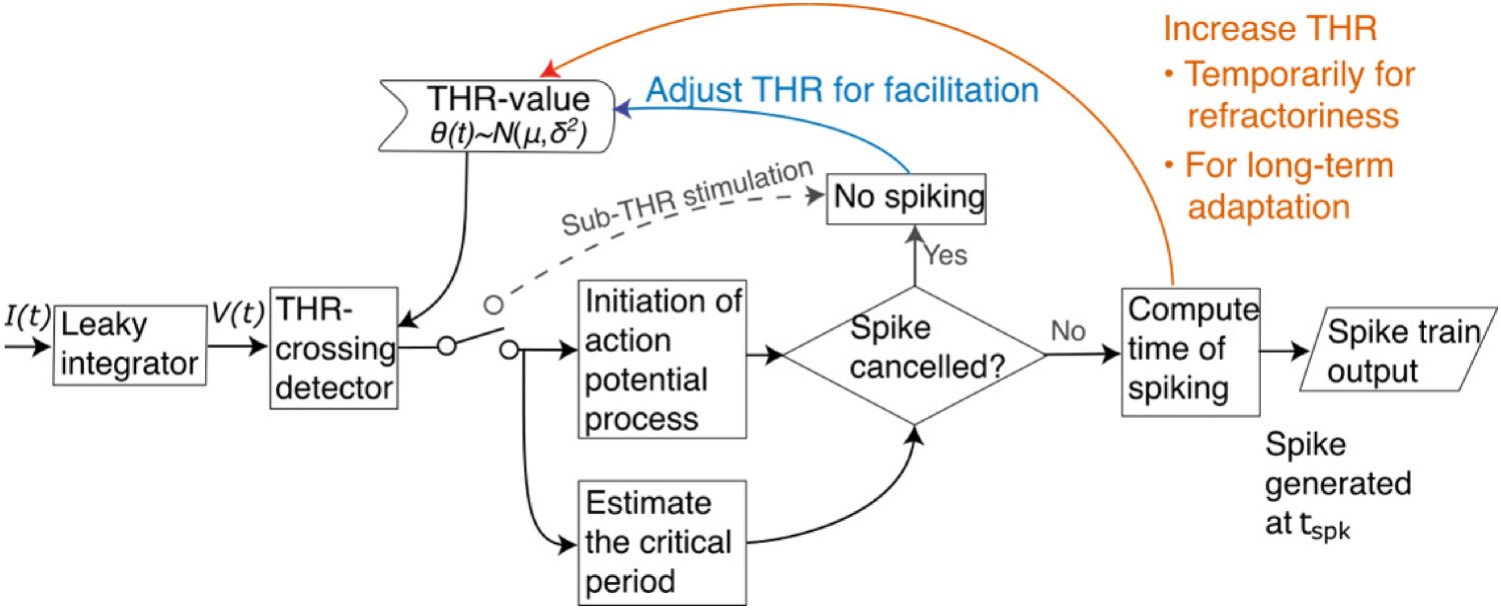
Flowchart of the present model that builds on the biphasic leaky integrate-and-fire (BLIF) model by Horne et al. (2016). The neuron is thought to integrate the incoming electrical current and to release an action potential if the capacitive membrane voltage reaches the stochastic threshold of the neuron and if the action-potential-initiation process is completed before the neuron is depolarized by the second phase of a charge-balanced biphasic pulse. Here, the BLIF model is extended for pulse-train stimulation by adding elements that simulate the refractoriness and adaptation phenomena after each spiking of the neuron, and one that models the active component of facilitation upon sub-threshold (THR) stimulation.

Figure 1 depicts the working principle of the present model for pulse train stimulation. The BLIF model (Horne et al., 2016) is used to integrate the incoming electrical current starting from either the beginning of the stimulus or the time of last spiking. Following the above-described steps, the next threshold crossing then launches the initiation of the action-potential process and determination of whether that process is completed in time before the lagging phase of a biphasic pulse repolarizes the neuron. A successful completion of the initiation process results then in inevitable spiking of the neuron at the time instant $t_{\mathrm{spk}}$ , at which the neuron's membrane voltage is also set to zero and the refractoriness and long-term adaptation processes are activated to increase the neuron's threshold value temporarily. On the other hand, every pulse that fails to evoke an action potential activates the so-called active component of facilitation in the model – reducing the neuron's threshold value temporarily so that subsequent pulses have a better chance of exciting the neuron to spike.

#### Refractoriness

The fundamental physiological mechanisms behind the refractory behavior of neurons are described in neurophysiological studies of which first ones date back over a century (Tait, 1910). Once a neuron has generated an action potential, its ion channels remain inactive for a while, preventing the neuron to be excited during the so-called absolute refractory period (ARP). Afterwards, the neuron gradually recovers to its resting state as more and more ion channels become active again. During this relative refractory period (RRP), the neuron can be excited, but its threshold is elevated. In electrically stimulated auditory nerve fibers, the absolute refractory period has been estimated to last for about 0.3 to 0.7 ms from the onset of the spike-evoking electrical stimulus (Dynes, 1996; Miller et al., 2001), whereas estimates for the duration of the relative refractory period range from 0.4 ms (Miller et al., 2001) to 5 ms (Imennov & Rubinstein, 2009) between studies.

Here, we followed a traditional approach for simulating refractory behavior of the ANF in phenomenological models (for a review, see Takanen et al., 2016). Upon a successful completion of the action-potential-initiation process, the threshold value of the modeled neuron is multiplied with an exponential function (similar to the one by Hamacher, 2003):

(2) $$\hat{\theta }(t)=\;\theta (t)\{\begin{array}{c} \begin{array}{c} \infty ,\;\;if\;t<t_{0}+t_{\mathrm{ARP},}\\ {\left[\left(1-\exp\left(\frac{-t+t_{\mathrm{ARP}}}{q\tau_{\mathrm{RRP}}}\right)\right)\left(1-r\exp\;\left(\frac{-t+t_{\mathrm{ARP}}}{\tau_{\mathrm{RRP}}}\right)\right)\right]}^{-1},\;\;if\;t\geq t_{0}+t_{\mathrm{ARP}}. \end{array} \end{array}$$

Here, the constants *q* and *r* were defined to have values of 0.76 and $\;8.77\times 10^{-3}$ , respectively, after least-square optimization of the model performance against neurophysiological data for refractoriness (below). The absolute refractory time $t_{\mathrm{ARP}}\;$ was fixed at 0.3 ms following Miller et al. (2001), while the time constant for the relative refractory period $\tau_{\mathrm{RRP}}$ is designed to vary in order to emulate noisiness of the ion flow into the neuron. Specifically, the normal distribution for $\tau_{\mathrm{RRP}}$ is designed to have an average value of 1.5 ms and a variance of 0.4 ms. Another noteworthy distinction between the present approach and previous ones is that, here, the absolute refractory period is considered to have begun when the action-potential process was initiated (i.e., at the time of threshold crossing $t_{0}$ ). If the absolute refractory period would be considered to begin only at the time of spiking, like in several phenomenological models (Campbell et al., 2012; Goldwyn et al., 2012; Hamacher, 2003; Joshi et al., 2017; Van Gendt et al., 2016), the effective absolute refractory period could become too long. That is because the spiking latency of the neuron is inherently included in the neurophysiological data, in which the refractory period has been defined starting from the onset of the preceding supra-threshold pulse (Cartee et al., 2000; Dynes, 1996; Miller et al., 2001).

#### Element for Long-Term Adaptation

In the case of a time-invariant pulse-train stimulation, the spiking activity of the ANF drops progressively over the duration of the stimulus. This drop exceeds what could be explained by refractoriness and is associated with spike-rate adaptation – the neuron's adaptation to time-invariant stimulation. Physiologically, spike-rate adaptation has been associated, e.g., with slow after-hyperpolarization (Gulledge et al., 2013) and activity of Kv1.1 and HCN channels that progressively shift the resting membrane potential (Boulet & Bruce, 2017; Mo et al., 2002; Negm & Bruce, 2014). To simulate such a progressive drop in responsiveness of the ANF in the present model, an element for long-term adaptation was designed to increase the threshold of the modeled neuron upon spiking beyond the effective duration of the refractoriness explained above. The element for long-term adaptation gets activated at the predicted time of spiking and the threshold of the neuron is elevated from that moment onwards, in addition to the incremental effect of the refractoriness-component, by multiplying the threshold value $\hat{\theta }(t)$ with a time-variant adaptation coefficient

(3) $$\begin{array}{c} A(t)=\hat{A}(t)\times \;min\left(m_{a},\;1+\;c_{a}\exp\;\left(-\frac{t}{t_{a}}\right)\right) \end{array}$$

that increases incrementally – from that moment onward – every time the neuron spikes. The adaptation coefficient A is initiated with ones and $\hat{A}$ denotes the old values of the adaptation coefficient, containing the incremental effects of the previous spiking activities on the values. Here, $t_{a}$ denotes a time constant having a value of 125 ms and $c_{a}$ is a normally distributed coefficient $\left(c_{a}∼N(.01,0.01\right)$ ), defining the initial increment to be one percent, on average. The constant $m_{a}$ , having a value of 1.38, is used here as an assumed upper limit on how much the neurophysiological mechanisms behind long-term adaptation can shift the neuron's resting potential (for a review, see Boulet et al., 2016). The aforementioned values for $t_{\mathrm{Adapt}}$ , $c_{\mathrm{Adapt}}$ and the limit for activation of the element were obtained by optimizing the model's performance against neurophysiological data about the variation in spiking activity during the stimulation (Miller et al., 2008) with a generic optimization algorithm (Conn et al., 1991). Our model introduces the concept of an upper limit for the effect size, but otherwise similar approaches and similar time constants have been used earlier to model long-term adaptation (Campbell et al., 2012; Nourski et al., 2006; Van Gendt et al., 2016; Van Gendt et al., 2017).

#### Active Component of Facilitation

**Figure 2.**
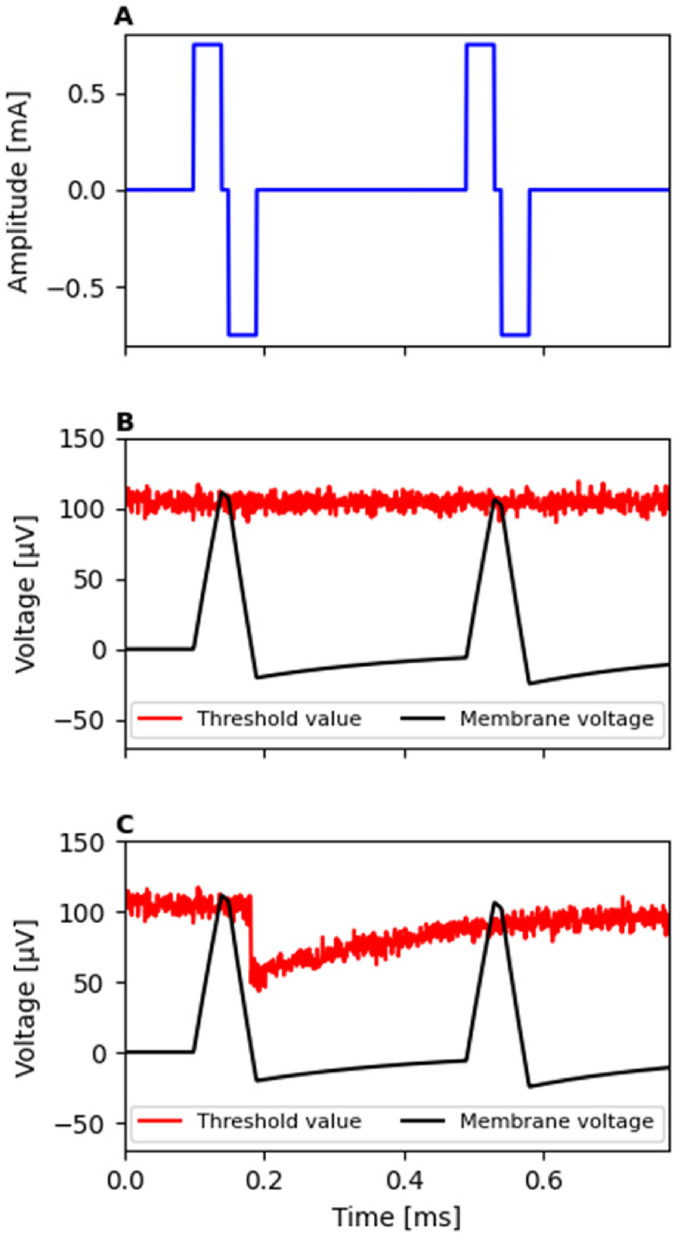
Example about how the active component of facilitation allows the model to account for facilitation with charge-balanced pulses. Due to the sub-threshold stimulus amplitude of the pulses presented (A) to the model, neither of the pulses would be normally sufficient to excite the modeled neuron to spike (B). (C) Active component of facilitation (equation (4)) reduces the threshold of the model temporarily upon offset of a sub-threshold stimulation, allowing the second pulse to evoke the modeled neuron to spike.

Thanks to the facilitation phenomenon (Lucas, 1910), a pulse that itself cannot excite the neuron to spike may enable the subsequent pulse to do so. Neurophysiological studies (for a review, see Boulet et al., 2016) have led to the idea that there exist two physical components that result in slight prolonged depolarization of the neuron, thanks to which a smaller-than-normal charge is required from the second pulse to excite the neuron. The first component is passive, arising from the capacitive membrane charging and leading to a residual charge when two monophasic pulses are presented at short inter-pulse intervals (Dynes, 1996). LIF models, such as the present one, can elegantly capture this aspect of facilitation when the time constant is chosen accordingly. By doing so, Joshi et al. (2017) could reproduce the facilitation data collected by Dynes (1996) with monophasic pulses. However, the passive component is not sufficient to explain observed facilitation with charge-balanced pulses (Cartee et al., 2000; Heffer et al., 2010) because the charge dispatches faster due to the charge-balancing phase of the pulse and, consequently, the charge may even be below the resting potential after the second pulse is presented. Therefore, there must be other physical mechanisms as well behind facilitation. The residual activity of the sodium channels has been found to lead into prolonged depolarization after subthreshold stimulation (Hodgkin, 1938) and is, therefore, a good candidate for the active component of facilitation (Boulet et al., 2016). To emulate such an active component of facilitation in the present model, the threshold value of the modeled neuron is temporarily decreased after the offset of a given stimulation by multiplying the threshold values with a third-order polynomial function

(4) $$\begin{array}{c} \hat{\theta }(t)=\;\theta (t)(a_{0}+a_{1}t+a_{2}t^{2}+a_{3}t^{3}),\; \end{array}$$

where the polynomial coefficients were fit to have the values of $1.3\times 10^{-9},$ $-2.42\times 10^{-6}$ , $1.68\times 10^{-3}$ and 0.51 after optimizing the performance of the model against the neurophysiological data by Cartee et al. (2000). A third-order polynomial was selected as it was the lowest-order polynomial capable of modeling the effects. As shown in Figure 2, the offset of a given stimulation is defined here as the time instant at which the membrane voltage V(t) crosses zero (i.e., the resting potential) after it had been pushed above or below it by the anodic- or cathodic-leading charge-balanced pulse, respectively. Such a definition elegantly circumvents the necessity of providing the model with additional information about the pulse shapes and/or the inter-pulse interval for modeling facilitation with charge-balanced pulses (Cohen, 2009e; Goldwyn et al., 2012).

## Evaluation of the Model Performance Against Neurophysiological Data

The ability of the BLIF model to reproduce ANF response characteristics from neurophysiological studies involving single-pulse stimulation was already verified by Horne et al. (2016). Hence, the experimental verifications are here restricted to temporal phenomena related to inter-pulse interactions and responses to time-invariant pulse-train stimulations. All simulations were conducted using the model with the same parameters.

### Refractoriness

**Figure 3.**
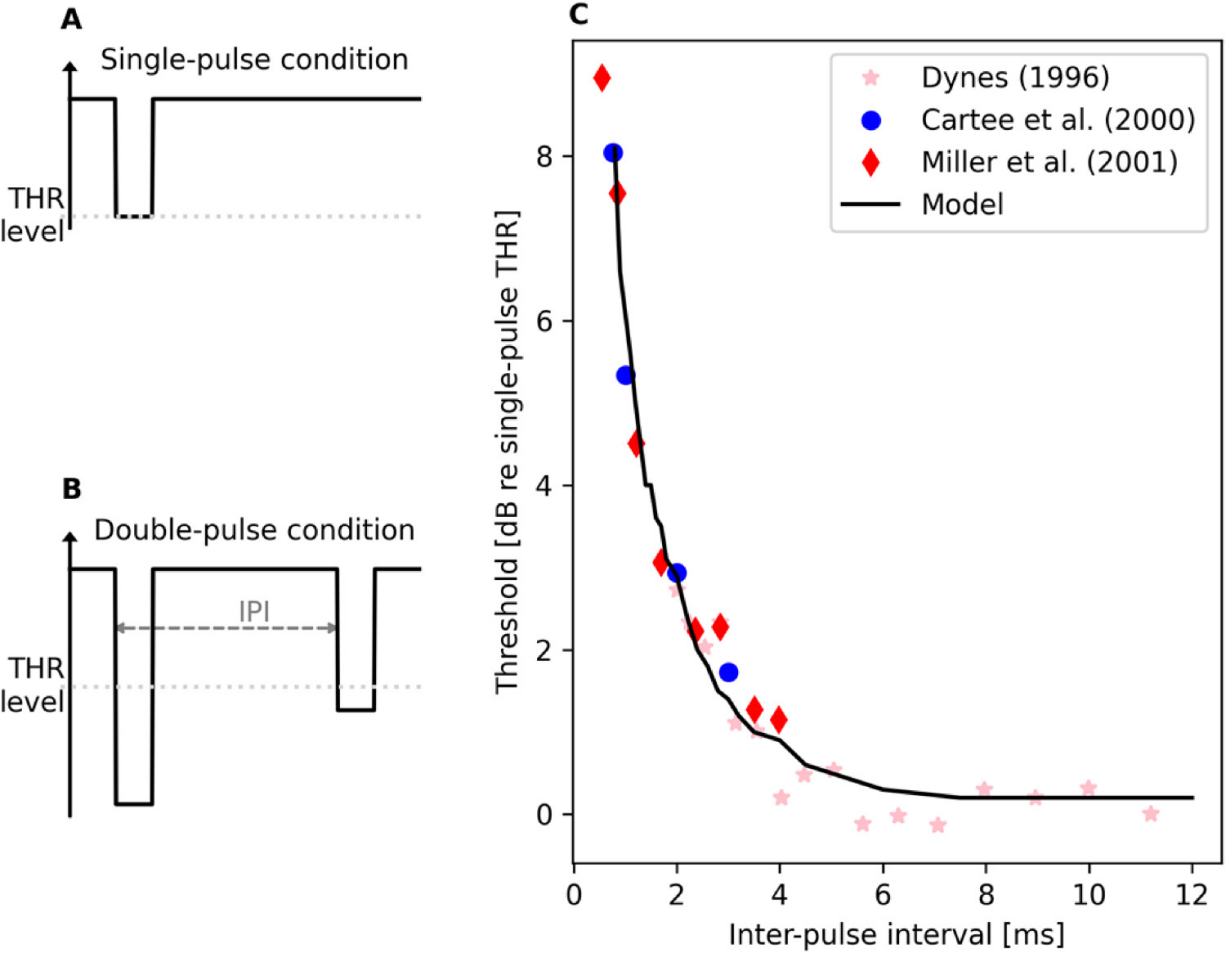
Results for modeling refractory recovery of the ANF after supra-threshold stimulation. The experimental paradigm used by Dynes (1996) was replicated by measuring the threshold values for the probe in single- (A) and double-pulse conditions (B). The results (C) show that the model reproduces the neurophysiological data (Cartee et al., 2000; Dynes, 1996; Miller et al., 2001).

Following the experimental paradigm used in the neurophysiological study by Dynes (1996), refractoriness characteristics of the model were evaluated by measuring threshold values for 40-µs-long monophasic pulses. In the single-pulse condition (Figure 3A), the probe pulse was presented in isolation and the threshold value obtained for this condition provided the reference to which the thresholds obtained in different paired-stimulus conditions (Figure 3B) were compared against. Specifically, the paired-stimulus condition consisted of a supra-threshold masker pulse preceding the probe pulse at an inter-pulse interval (IPI) ranging from 0.5 ms to 12 ms.

In both conditions, the threshold value was determined simply as the stimulation level for which the model predicted 50% spiking probability to the probe based on 500 iterations at each level. It should be noted that cases where the model did not spike to the supra-threshold masker (presented at a level corresponding to 90% single-pulse spiking probability) were excluded from the threshold analysis for the paired-stimulus condition. The threshold for a given paired-stimulus condition was determined as undefinable if 50% spiking probability to the probe was not achieved at stimulation levels 20 dB above the single-pulse threshold value. Results in Figure 3C show that the model output prediction matches with the neurophysiological data (Cartee et al., 2000; Dynes, 1996; Miller et al., 2001).

### Facilitation

**Figure 4.**
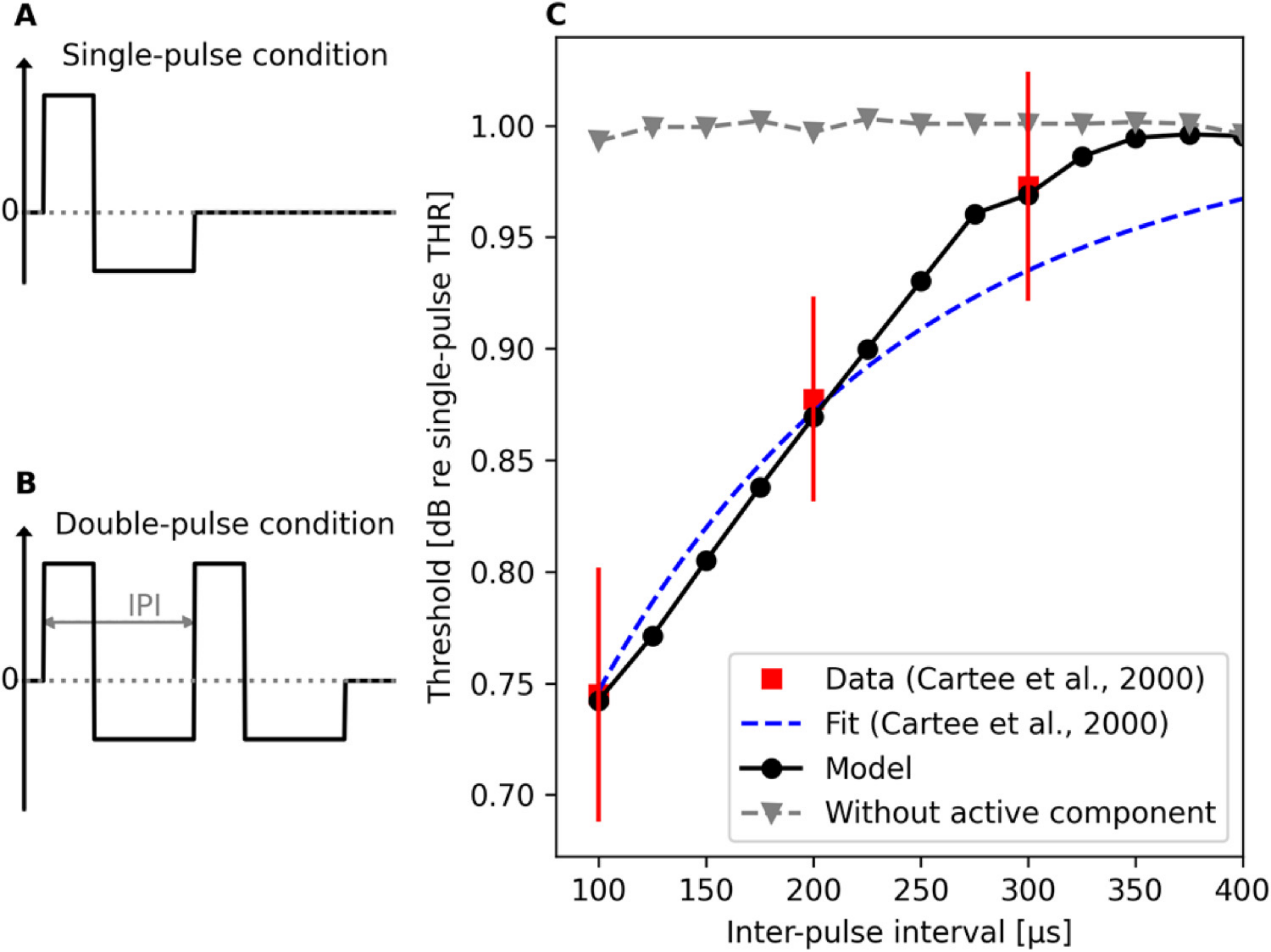
Results for modeling facilitation with charge-balanced pseudo-monophasic pulses following the experimental paradigm used by Cartee et al. (2000). The amount of facilitation was determined based on the difference in the threshold values between the single- (A) and double-pulse conditions (B). The model output (C) predicts the observed temporal decrease in threshold at short inter-pulse intervals (Cartee et al., 2000). Results from modeling the facilitation without the active component of facilitation are shown as well, illustrating the necessity of the component in the model.

Here, the interest was placed on investigating facilitation with charge-balanced pulses that are used in cochlear implants to avoid harmful effects caused by net flow of current. Suitable single-fiber data were obtained by Cartee et al. (2000) in their neurophysiological study with deafened cats and that study was, therefore, chosen for simulation. Following the experimental setup by Cartee et al. (2000), pseudo-monophasic pulses were presented to the model in both single- (Figure 4A) and double-pulse conditions (Figure 4B), the two pulses being identical in the double-pulse condition. Similar to the analysis for evaluating refractoriness, the difference in the threshold values between the single- and double-pulse conditions was computed and used to determine the amount of facilitation.

Here, facilitation was evaluated at IPIs ranging from 100 µs to 500 µs (with a step size of 25 µs) using 100 iterations per each IPI and stimulation level to determine the level resulting in 50% spiking probability. To illustrate the importance of the active component of facilitation in the model, simulations were performed also with that component being disabled in the model. It should be noted that, like in the neurophysiological studies by Cartee et al. (2000), the pseudo-monophasic pulse had always a 50-µs-long leading excitatory phase but the level & duration of the second charge-balancing phase depended on the IPI between the two pulses in the double-pulse condition, as illustrated in Figure 4A and 4B. Hence, also a unique single-pulse threshold value had to be determined also for each pseudo-monophasic pulse used in the double-pulse condition. Results in Figure 4C illustrate the model prediction to match with the neurophysiological data, but only when the active component of facilitation is enabled. This is not that surprising because, as stated above, the parameters of the active component of facilitation were selected for optimized performance of the model in this experiment. Therefore, we used also another experiment (below) to evaluate the model performance in terms of facilitation.

### Facilitation and Accommodation with Pulse Trains

**Figure 5.**
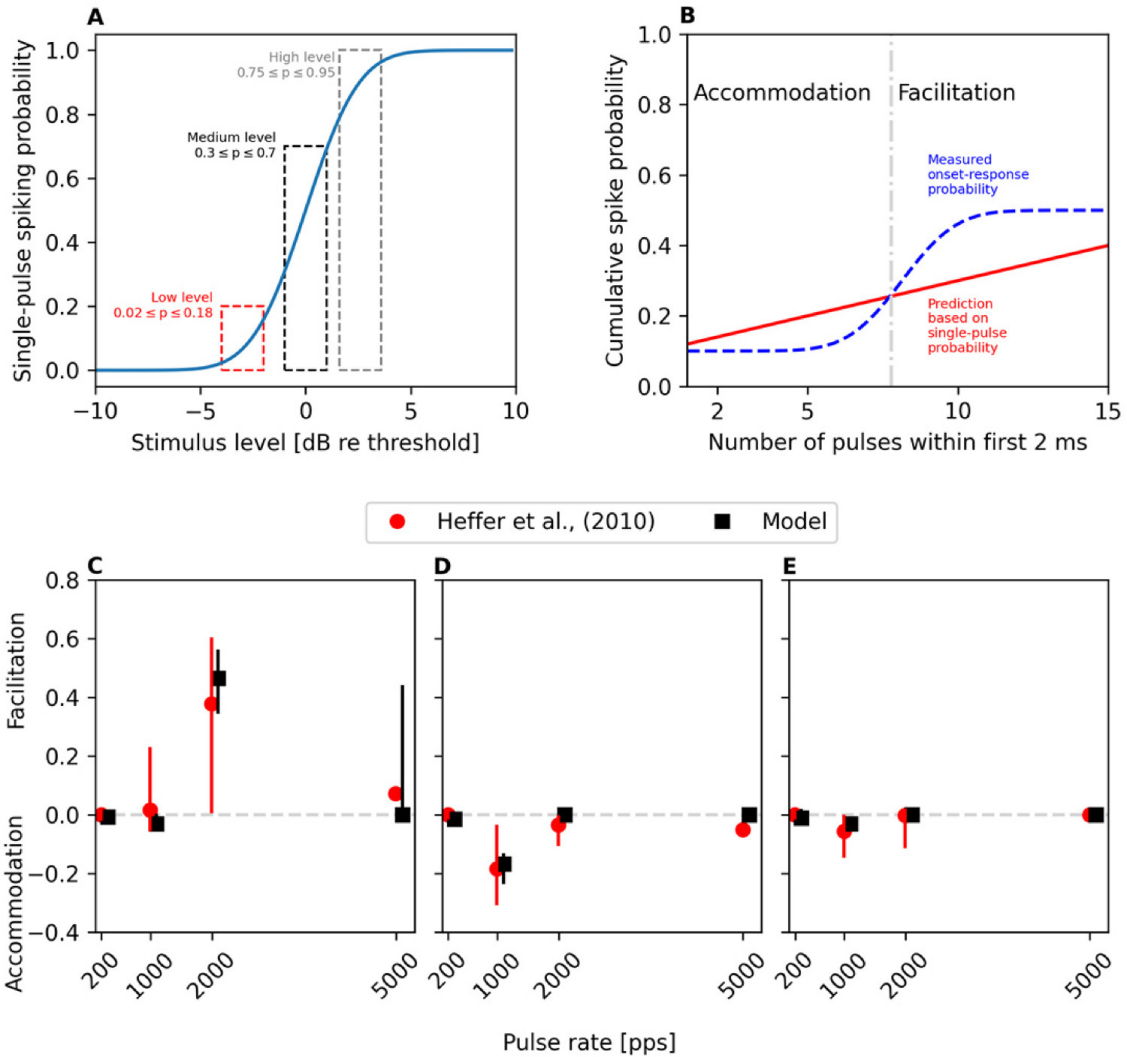
Following Heffer et al. (2010), facilitation/accommodation was assessed with sequences of charge-balanced biphasic pulses in terms of change in the onset-spiking probability during the first 2 ms of the pulse train sequence. (A) Three different stimulation level regions were selected based on the single-pulse spiking probability. (B) The underlying assumption in their experiment was that the cumulative probability of spiking would depend linearly on the number of pulses within the 2-ms-long time frame if no accommodation or facilitation would occur. In other words, observed spiking probabilities exceeding / falling below the linear prediction were interpreted as sign of facilitation / accommodation, respectively. The median values and quartile-ranges depicted in (C-E) show how the model reproduces the data by Heffer et al. (2010) – facilitation occurs at low stimulation levels (C), especially with 2,000 pps pulse train, while accommodation occurs at intermediate pulse rate (1,000 pps) with medium stimulation levels (D) and to a smaller degree at the high stimulation levels €.

Heffer et al. (2010) investigated accommodation and facilitation with 300-ms-long pulse trains consisting of biphasic pulses (25 µs per phase, 8 µs IPG) presented at rates of 200, 1,000, 2,000 and 5,000 pps. The pulse trains were presented at levels that yielded in desired single-pulse spiking probabilities. As depicted in Figure 5A, the low-stimulation levels targeted for probabilities between 0.02 and 0.15, the medium-stimulation levels for probabilities between 0.35 and 0.55, while probabilities between 0.7 and 0.9 were strived for with the high-stimulation levels.

Accommodation / facilitation was then assessed in terms of onset-response probability, which Heffer et al. (2010) defined as the probability of observing at least one spike within the first 2 ms of the given pulse-train sequence. The measured onset-response probability was thus obtained by presenting the 200, 1,000, 2,000 or 5,000 pps pulse train sequence at the given presentation level (Figure 5A) repeatedly and counting how many times (among the iterations) the pulse train elicited at least one spike within the first 2 ms from the stimulus onset. The measured probability was then compared to a linear prediction based on the single-pulse spiking probability and number of pulses within the time period. The linear prediction in Heffer et al. (2010) was made based on the assumption that the ANF would respond independently to each pulse in the sequence, ignoring also effects of refractoriness. Following this assumption, they obtained onset-response probability predictions straightforwardly by multiplying the single-pulse probability (Figure 5A) with the number of pulses within the first 2 ms in the given pulse-train sequence (Figure 5B). Figure 5B depicts how the difference between the measured and predicted onset-response probabilities was then interpreted as evidence for accommodation or facilitation depending on whether the difference, denoted as spike probability change, was found to be negative or positive, respectively (Heffer et al., 2010).

Here, the experimental design and analysis performed by Heffer et al. (2010) was followed by presenting identical pulse-train sequences to the model. For each singe-pulse spiking probability level (Figure 5A), 21 stimulation levels were included to cover the given probability range. The S-BLIF model was used to process the pulse trains 100 times at each stimulation level. Then the average onset-response probability was computed per stimulation level across the 100 iterations. This average onset-response probability was then compared against the prediction (Figure 5B) based on the single-pulse spiking probability the given stimulation level corresponds to, resulting in a difference value between the model output and the prediction (Figure 5B). Finally, the median and quartile values of the differences were computed across the stimulation levels corresponding to the given single-pulse spiking-probability range (Figure 5A) in order to obtain comparable data to the values reported by Heffer et al. (2010). Figure 5C–E illustrates that the model reproduces the neurophysiological data at all stimulation levels – exhibiting facilitation at higher pulse-rates, mostly at low stimulation levels (Figure 5C), and accommodation at 1,000 pps especially at medium stimulation level (Figure 5D).

It should be noted that the way facilitation and accommodation were assessed in Heffer et al. (2010) has a conceptual issue that biases the observed amount of facilitation and/or accommodation in their results and similarly also in the modeled results. Considering for instance the 1,000 pps pulse train, which has two pulses within the first 2 ms, the true onset response probability is

$$p_{o}=p^{2}+2(\;p(1-p))=2p-p^{2}$$

and not 2p that results from their linear prediction. Here, *p* denotes the single-pulse probability and the first term ( $p^{2}$ ) the probability that both pulses excite the neuron and the second term (p(1−p)) denotes the probability that either the first or the second pulse excites the neuron but the other one does not. Hence, the linear prediction will overestimate the true probability, leading to an overestimation of the amount of accommodation. Since the approach of Heffer et al. (2010) was followed here to the point, the bias introduced by the methodology does not change the conclusion that the model is able to reproduce their data. Nevertheless, another study was replicated in order to verify the degree the model reproduces the accommodation phenomenon for charge-balanced pulses.

**Figure 6.**
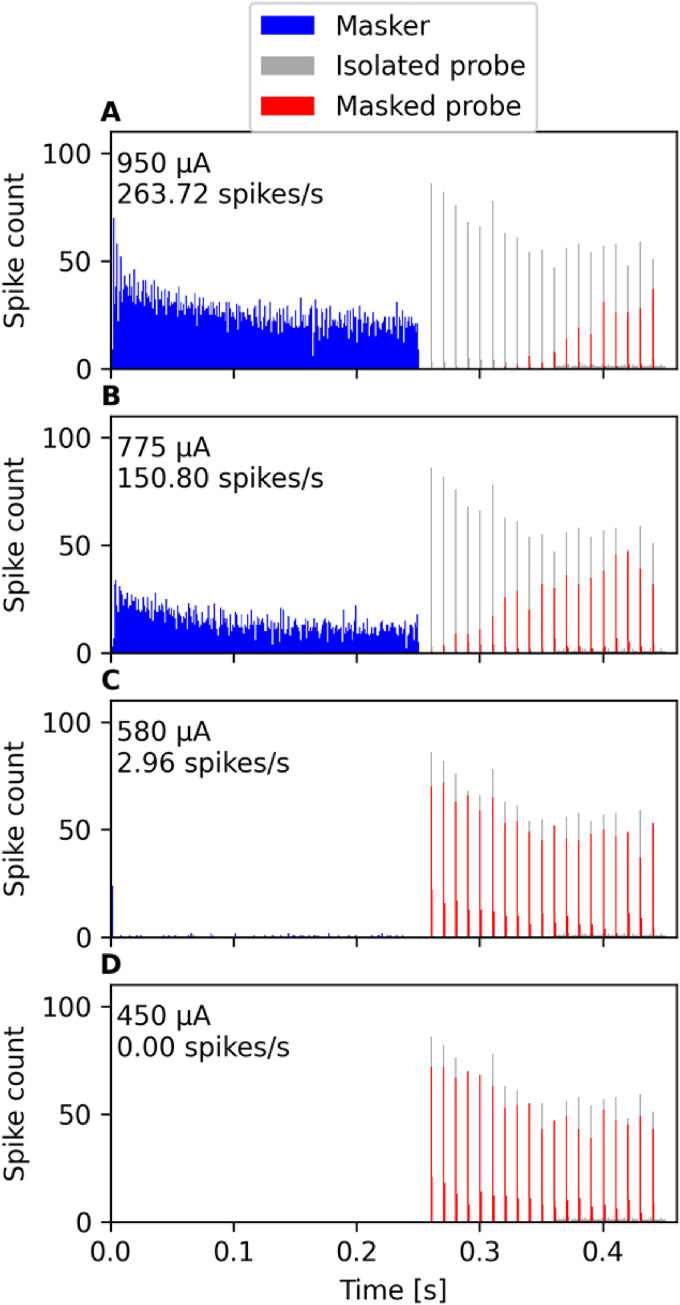
Following Miller et al. (2011), accommodation with pulse train sequences was inspected also in terms of how much a preceding 250-ms-long masker pulse train affects the responsiveness of the ANF to a 200-ms-long probe pulse train at different masker levels. The panels A-D show post-stimulus histograms for both the masker (5,000 pps) and probe (100 pps) pulse trains, when the probe is presented either in isolation or after a masker whose stimulation level varies. The responsiveness of the modeled neuron for the 100 pps probe pulse train is predicted to increase as the masker level decreases.

**Figure 7.**
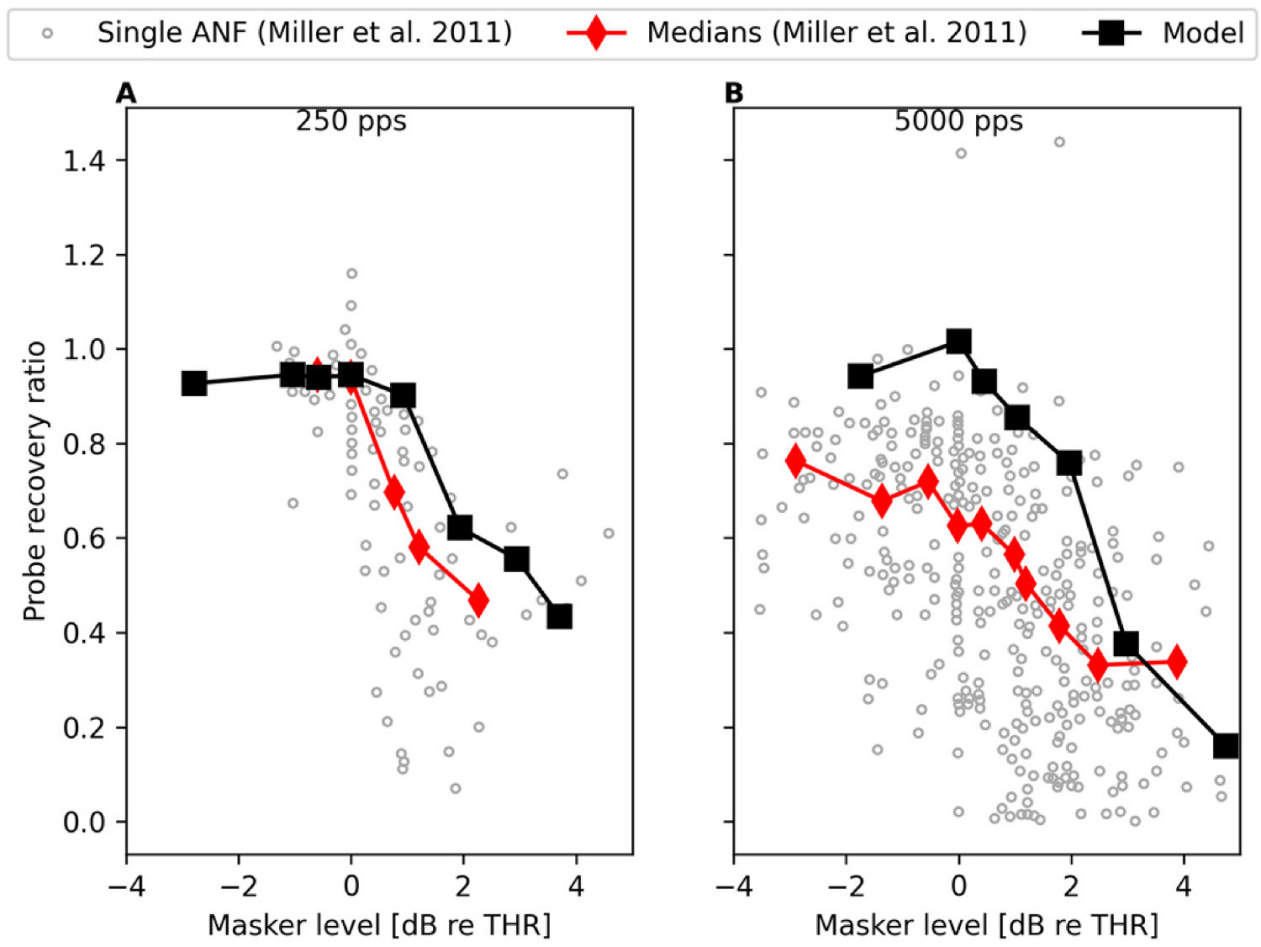
Results from modeling the amount of accommodation with pulse train sequences following Miller et al. (2011). The model predicts the accommodation effect for supra-threshold masker levels but does not reproduce the subthreshold response reduction observed by Miller et al. (2011) for a 5,000 pps masker (panel B).

Miller et al. (2011) investigated accommodation also with pulse train sequences but from the perspective of how much the spiking activity evoked by a preceding (masker) pulse train affects the spiking activity of the subsequent probe pulse train. A 250-ms-long 100 pps pulse train was used as the probe and its level was kept fixed at the level for which the probe achieved 30–70% spiking efficiency (spikes/pulses) when presented in isolation. The pulse rate of the masker pulse train was either 250 or 5,000 pps and the masker length was set at 200 ms. The masker level was varied and the results from different fibers were brought to the same scale by representing the stimulation levels in dB in respect to the stimulation amplitude at which the masker evoked at least one spike for the given ANF. Miller et al. (2011) found that the preceding masker pulse train reduces the spiking activity evoked by the probe pulse train even when the masker itself elicits only few or no spikes at all. They used the probe recovery ratio to quantify how well the responsiveness of the ANF to the probe pulse train is affected by the preceding masker pulse train. Here, we used the model to replicate the study, using the same pulse train sequences consisting of the same biphasic pulses (40 µs phase duration, 30 µs IPG) and keeping the presentation level for the probe pulse train fixed at the level, with which the probe achieved approximately 67% spiking efficiency. The post-stimulus histograms in Figure 6 show that the model predicts the 5,000 pps masker pulse train to reduce the spiking activity evoked by the probe pulse train also when the masker itself barely excites the neuron to spike. Inspection of the predicted probe recovery ratios, shown in Figure 7, reveals that the model predicts the accommodation effect for supra-threshold masker levels but does not reproduce the effect observed by Miller et al. (2011) that the 5,000 pps masker pulse train reduces the spiking activity evoked by the probe also at subthreshold masker levels.

### Spike-Rate Adaptation

To demonstrate effects of spike-rate adaptation on the responsiveness of the electrically stimulated ANF, Javel (1990) measured the spiking efficiency (i.e., spikes/pulses) at various pulse rates as a function of the stimulation current. He showed that a similar increase in stimulation current results in a more substantial increase in spiking efficiency at low pulse rates than at high pulse rates. In other words, a more substantial increase in stimulation current is required at higher rates to increase the spiking activity of the neuron above its preferred rate.

**Figure 8.**
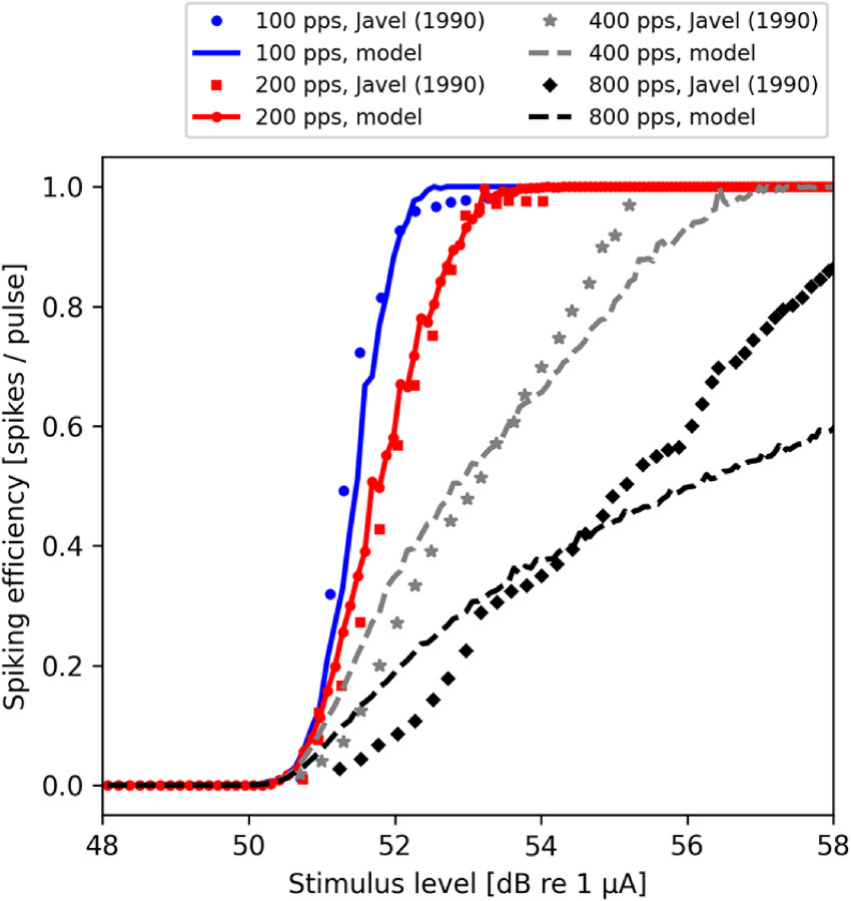
Following the experiments by Javel (1990), 100-ms-long pulse trains of biphasic pulses (50 μs phase and gap duration) were simulated with the model. The graph shows the original data from Javel (1990) as individual symbols and the model predictions. Here, an offset of −7.2 dB (re 1 µA) has been introduced to the model predictions in order to account for a general difference between the predictions and the neurophysiological data. The curves reproduce the trend of decreasing growth of spiking efficiency at higher pulse rates seen in the neurophysiological data, also at 800 pps at least up to 50% spiking efficiency.

To simulate the experiments by Javel (1990), 100-ms-long pulse trains of biphasic pulses (50 µs phase and gap duration) were simulated with the model. One hundred simulations were done at each pulse rate (100, 200, 400 and 800 pps) at a given stimulation level (from 600 to 1,400 µA in 10 µA steps) and the average spike counts were computed to derive estimates about the spiking efficiency at a given stimulation level. The results are shown in Figure 8 together with the neurophysiological data by Javel (1990). There, an offset of −7.2 dB (re 1 µA) has been introduced to the model predictions in order to account for the general difference between the predictions and the neurophysiological data. In overall, the model can be seen to reproduce the trends in the neurophysiological data: The model predictions match well with the neurophysiological data at low pulse rates (100 and 200 pps). For the highest pulse rate(s), the model tends to overestimate the drop in spiking efficiency at the highest stimulation levels, but even at the highest tested rate of 800 pps it still reproduces the results well up to 50% spiking efficiency.

Inspection of the spike timings and the inter-spike intervals provides another angle to the spike-rate adaptation phenomenon. Due to the adaptation phenomenon, the ANF response manifests an oscillatory pattern with alternating periods of higher spiking activity and lower spiking activity (Heffer et al., 2010). Consequently, spikes are recorded at integer multiples of the inter-pulse interval. This aspect of spike-rate adaptation was demonstrated by Miller et al. (2008) who inspected spiking activity of 88 fibers within specific time frames (0–12 ms, 4–50 ms and 200–300 ms from the stimulus onset) upon repetitive (30 to 100 iterations) stimulation with biphasic pulse trains (40 µs per phase) of 250, 1,000 and 5,000 pps. Here, we simulated the experimental conditions in Miller et al. (2008) and presented 300-ms-long pulse trains of 250, 1,000 and 5,000 pps (40 µs per phase, 10 µs IPG) 100 times to the model. Stimulation levels for the 250, 1,000 and 5,000 pps pulse trains were set to 1,110, 1,200 and 1,100 µA, respectively. For each spike-train output of the model, we computed the inter-spike intervals between consecutive spikes within the 4–50 ms analysis window from the stimulus onset as in Miller et al. (2008). The values from the 100 iterations were pooled together. We then performed a similar histogram analysis of the inter-spike intervals as done by Miller et al. (2008) using also 50-µs-wide bins. The bin heights were normalized to enable direct comparison between the model performance against the neurophysiological data collected by Miller et al. (2008) for 250, 1,000 and 5,000 pps pulse trains at stimulation levels of 1,150, 1,300 and 1,200 µA, respectively. In addition, Pearson-correlation analysis was performed to evaluate the similarity between the model output and the neurophysiological data in a quantitative manner.

**Figure 9.**
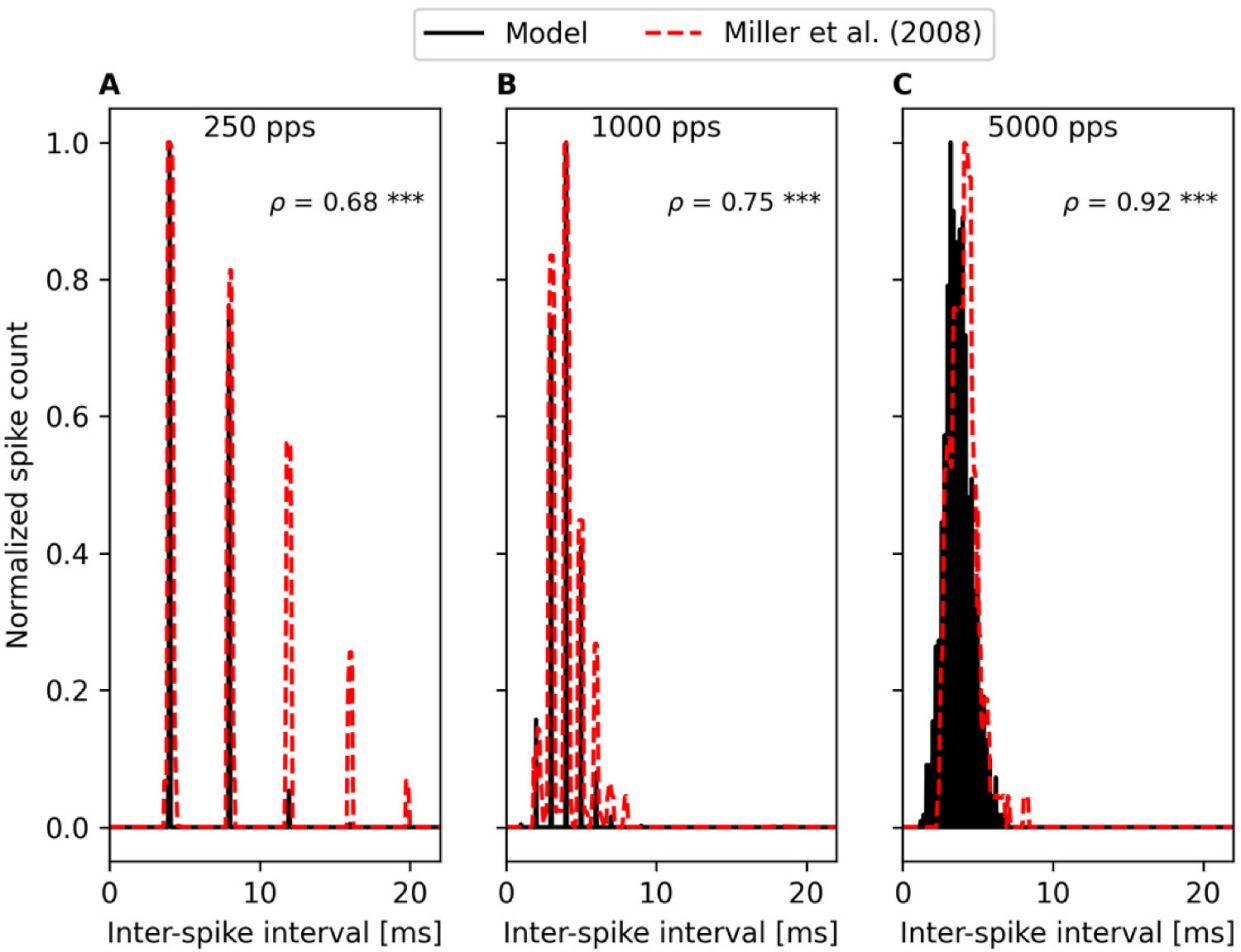
The effect of spike-rate adaptation on spike timings was evaluated for time-invariant pulse trains by inspecting inter-spike intervals. Spiking was evaluated within 4 to 50 ms after the stimulus onset for 300-ms-long pulse trains following Miller et al. (2008). The model output exhibits high correlation with the original data (p < 0.001 for all). The regular spiking at integer multiples of the inter-pulse interval is demonstrated at 250 (A) and 1,000 pps (B). At 1,000 pps (B), the highest peak does not occur at the inter-spike interval corresponding to the pulse rate but at 4 ms because refractoriness limits the spiking at shorter inter-spike interval. Both data and model prediction exhibit a stochastic distribution of spiking intervals at the highest pulse rate of 5,000 pps (C).

Results in Figure 9 show the model predictions to match with the neurophysiological data, with a high correlation (p < 0.001) for all pulse rates. For 250 pps (Figure 9A), the periodic distribution of responses at integer multiples of the IPI is reproduced. The extent of adaptation is slightly smaller in the prediction as the peaks are slightly smaller at larger inter-spike intervals. At 1,000 pps, spikes are generated still at integer multiples of the IPI but refractoriness limits the responsiveness at the shortest IPIs and, therefore, both data and model predictions show the highest peak around 4-ms inter-spike interval (Figure 9B). At 5,000 pps, both the data and the model prediction exhibit a stochastic distribution of spike timings and the reduced synchrony between pulse-train input and spike-train output (Figure 9C).

### Temporal Coding

All above-mentioned temporal phenomena affect how accurately the information carried by the pulse-train stimulus is conveyed in the spiking activity of the auditory nerve fiber. Analysis of synchronization between the input and output signals provides a convenient and therefore often-used tool for assessment of that accuracy. It has been well established in neurophysiological studies that electrical stimulation of the ANF results in better synchronization than what can be achieved in acoustical stimulation (see, e.g., Hartman & Klinke, 1990; Moxon, 1971). The synchronization is high at low pulse rates (until about 800 pps) and decreases then at higher pulse rates, the extent of the decrease varying between neurophysiological data.

**Figure 10.**
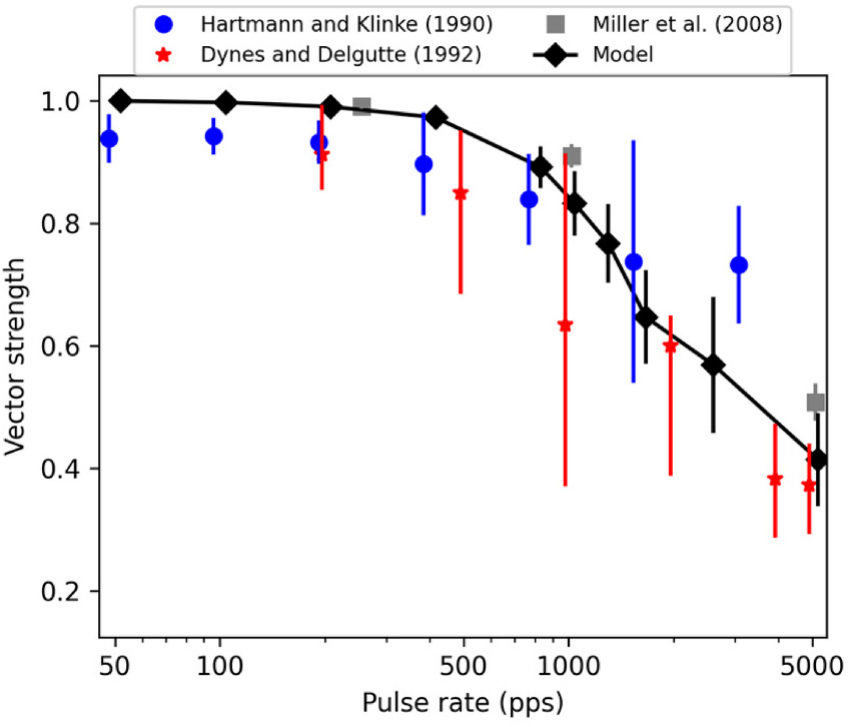
Vector-strength values computed between biphasic (40 μs per phase, 30 μs IPG) pulse-train inputs and spike train outputs provided by the model. For comparison, neurophysiological data from both sinusoidal (Dynes & Delgutte, 1992; Hartman & Klinke, 1990) and pulsatile electrical stimulation of cat auditory nerve fibers (Miller et al., 2008) are shown. The error bars denote 95% confidence intervals for the data by Dynes and Delgutte (1992); Hartman and Klinke (1990), and standard deviations for the Miller et al. (2008) data and model predictions. Both the model predictions and the neurophysiological data show very high synchrony at low stimulation rates and a gradual decrease of synchronization above 800–1,000 pps. The extent of the decrease varies slightly across neurophysiological studies. However, the trend is reproduced by the model.

In order to evaluate how well the model performance matches with the neurophysiological data, vector-strength values (Goldberg & Brown, 1968) were computed from the model outputs for 300-ms-long pulse trains of biphasic pulses (40 µs per phase, 30 µs IPG) at various rates (50, 100, 200, 400, 800, 1,000, 1,250, 1,600, 2,500 and 5,000 pps). Selection of stimulation level is arbitrary (and not generally reported in neurophysiological studies) but has together with the pulse shape a significant impact on the number of evoked spikes and consequently, on the vector-strength values. Here, the model was simulated at a level of 767 µA (corresponding to 90% spiking probability for a single pulse) at pulse rates up to 1,600 pps. At 2,500 and 5,000 pps, stimulation levels of 782 and 797 µA were used, respectively, to ensure that the spiking rate lies, on average, between 240 and 310 spikes/s – as in the experimental data by Miller et al. (2008). One hundred simulations were performed at each pulse rate, and the average and standard deviations of the resulting vector-strength values were computed to predict the synchronization at the given pulse rate. The resulting values are shown in Figure 10 along with the neurophysiological data from sinusoidal electrical stimulation of cat ANFs (Dynes & Delgutte, 1992; Hartman & Klinke, 1990) as well as from pulsatile electrical stimulation of cat ANFs (Miller et al., 2008). Both neurophysiological data and model predictions show a high degree of synchronization up to about 800 pps (or 800 Hz rate in sinusoidal stimulation) and then gradually reducing synchronization at higher stimulation rates. The model prediction is in better agreement with the steeper decrease of synchronization (Dynes & Delgutte, 1992; Miller et al., 2008) while the data by Hartman and Klinke (1990) exhibit a shallower decrease towards higher stimulation rates.

## Discussion

This work presents a phenomenological model for the auditory nerve fiber's (ANF's) response to pulse train stimulation based on single-fiber cat ANF data from literature. Building on the biphasic leaky integrate and fire (BLIF) model by Horne et al. (2016), the modeled ANF is thought to integrate the incoming electrical current and to release an action potential if the membrane voltage reaches the neuron's stochastic threshold and if the neuron is not repolarized before it is ready to spike. To that end, the action potential process is divided into two separate processes: an initiation process and a computation of the time of spiking, of which the former has to be finished within a critical period (Rubinstein et al., 2001). If the initiation process is completed in time, the spike's latency and jitter are computed based on how greatly the threshold is exceeded. In the present work, we extend that model for pulse train stimulation by adding elements that simulate refractoriness, facilitation and long-term adaptation. Specifically, elements for refractoriness and long-term adaptation are designed to temporarily increase the threshold value of the modeled neuron after an action potential has been released. Refractoriness imposes a more substantial effect on the threshold, preventing the neuron to be excited during the absolute refractory period, while the smaller effect of the long-term adaptation extends over a longer period. On the other hand, the element for facilitation reduces temporarily the threshold value after sub-threshold, non-excitatory stimulation in order to facilitate the subsequent pulse to excite the neuron – emulating thus the active component of facilitation (Boulet et al., 2016; Hodgkin, 1938). By optimizing the functionality of the added elements, the overall complexity of the model is kept at a low level in order to ensure that the model can be conveniently tuned for individual CI users.

Here, we demonstrate the versatile ability of the S-BLIF model to reproduce neurophysiological cat single-fiber data from literature. Despite having only one integrator and a limited parameter space, the model can employ the same set of parameters and yet quantitatively reproduce data from different research labs employing diverse pulse shapes. It should be noted that similar aspects (or subsets of those) have been previously reproduced by other models as well. The S-BLIF model distinguishes itself by being able to reproduce diverse data related to refractoriness, facilitation, accommodation, spike-rate adaptation and temporal coding in quantitative terms with the same parametrization. Refractoriness is the most well-known amongst these phenomena and two of the here employed data sets (Dynes, 1996; Miller et al., 2001) have been used for fitting of the refractoriness function in several phenomenological models (Takanen et al., 2016). Still, according to the knowledge of the authors, only Joshi et al. (2017) have quantitatively evaluated their model performance using the data collected by Dynes (1996). Here, we show that the present model can reproduce also the data collected by Cartee et al. (2000) and by Miller et al. (2001), in both of which pronounced effects of refractoriness were observed at shorter inter-pulse intervals than what Dynes (1996) used.

The opposing effects of facilitation and accommodation have been of modeling interest before as well. Cohen (2009e) was able to reproduce facilitation and accommodation (as well as refractoriness) data from his ECAP (electrically evoked compound action potential) measurements with CI users in a qualitative manner. However, his approach requires prior knowledge about the inter-pulse intervals and the level of the pulses. Joshi et al. (2017) reproduced facilitation and accommodation data from recordings with monophasic pulses (Dynes, 1996) without a priori information about the stimulation conditions. Here, the focus was placed on facilitation and accommodation effects in stimulation with charge-balanced pulses because effects related to such pulses are more pertaining for modern CIs. The present model was shown to reproduce the facilitation effects observed by Cartee et al. (2000) with charge-balanced pseudo-monophasic pulses. Using the same set of parameters, the model reproduces also the facilitation data measured by Heffer et al. (2010) with trains of symmetric biphasic pulses. The accommodation effects that Heffer et al. (2010) observed at lower pulse rates are reproduced as well, mainly because of the charge of the leaky integrator recovering to the resting voltage only after the effect of the facilitation component has already ended. This aspect of the model enables the model to account also partially for the accommodation data collected by Miller et al. (2011), where the model correctly predicts the preceding masker pulse train to reduce the spiking activity evoked by the probe pulse train even when the masker pulse train itself evokes only few spikes. The effect of the leaky integrator is not sufficient to account for the finding in Miller et al. (2011), that the 5,000 pps masker pulse train can reduce the responsiveness of the neuron to the probe pulse train even when the masker is presented at subthreshold stimulation levels. A separate element for accommodation would be needed to account for that and the accommodation observed by Dynes (1996) for monophasic pulses.

Considering spike-rate adaptation, we followed previous modeling studies (Bruce et al., 1999, 2000; Goldwyn et al., 2012; Hamacher, 2003; Joshi et al., 2017; Van Gendt et al., 2016) and compare our model's output against the data from Javel (1990) and Miller et al. (2008). Despite the popularity of Javel’s (1990) data, the effects of pulse rate on the spiking efficiency have so far been demonstrated only qualitatively. Here, we demonstrated the capability of the present model to reproduce those effects in a more quantitative manner – by using the same stimulation parameters (apart from a small global offset in stimulation level) and by presenting the results in the same graph to ease the evaluation. The variation of stimulation levels and analysis windows used by Miller et al. (2008) makes it somewhat difficult to evaluate the performance of models so that the results bring across the effects of spike-rate adaptation. Perhaps because of this, both Goldwyn et al. (2012) and Joshi et al. (2017) analyzed the spiking activity of their model for the whole stimulus and compared the results against the data (Miller et al., 2008) that were recorded within a unique analysis window for each pulse rate. Both Goldwyn et al. (2012) and Joshi et al. (2017) were able to qualitatively demonstrate the effects of spike-rate adaptation on the inter-spike-interval histograms. However, the differences in stimulus characteristics and analysis hinder the assessment of their models’ performance. A more extensive evaluation was performed by Van Gendt et al. (2016), who inspected the spiking activity of their model within all three analysis windows and plotted the results side-by-side with the data from Miller et al. (2008) at various stimulation levels. However, only qualitative comparisons were made and a different pulse shape was used. Here, we wanted to perform a quantitative evaluation of the model performance against the neurophysiological data, mimicking the stimulation characteristics and using the same analysis window (4–50 ms from stimulus onset) in which the neurophysiological data exhibit clear examples of spike-rate adaptation for all pulse rates. By limiting the evaluation to a single analysis window and a single stimulation level per pulse rate, we were able to quantify the high correlation between the model prediction and the neurophysiological data.

Analysis of the vector strength values at different stimulation rates demonstrated that the present model replicates the effect of stimulation rate on the synchronization between the input sequence and the spike train output. Specifically, both the neurophysiological data and the model predictions were shown to exhibit high degrees of synchronization at rates up to about 800 pps, after which the synchronization drops gradually in agreement with the neurophysiological data by Dynes and Delgutte (1992) and Miller et al. (2008). When comparing the predictions of the present model to the ones of existing models, the model by Joshi et al. (2017) seems to predict a shallower decrease of synchronization following qualitatively the neurophysiological data by Hartman and Klinke (1990). In contrast, the present model seems to be in better agreement with the data by Dynes and Delgutte (1992) and Miller et al. (2008) from sinusoidal and pulsatile stimulation of ANFs, respectively.

Neurophysiological measurements have revealed insights into ANF's preferred & potential site(s) of excitation upon electrical stimulation. Van Den Honert and Stypulkowski (1984) observed differences in ANF response patterns at different stimulation intensities and postulated that near-threshold stimulation is more likely to excite the peripheral (dendritic) part of the ANF while the site of excitation shifts to the central (axonal) parts at higher stimulation intensities. By investigating the effects of pulse polarity on electrically-stimulated cat ANFs, Miller et al. (1999) found that cathodic-leading pulses were able to excite the neuron at lower stimulation levels than their anodic-leading counterparts but with the expense of longer latency. Together with the findings by Van Den Honert and Stypulkowski (1984), the findings from Miller et al. (1999) suggest that peripheral parts of the cat ANF would be more sensitive to the cathodic pulses while the neuron's central part would be more sensitive to anodic pulses. The opposite effect of pulse polarity on ANF thresholds and latencies was found in guinea pigs (Miller et al., 1998), which makes it difficult to generalize the phenomenon for all mammal species. Nevertheless, such findings bring motivation for more complicated model structures, having separate units to emulate the peripheral and central sites of excitation. In Takanen and Seeber (2016), we have indeed presented such a model where the two units were independently building up their membrane voltages to spike upon, while the principle of first-come, first-served was implemented, allowing only the earlier spike to be added to the output of the model and to reset both units into their refractory status. In a way, that model was similar to the later model by Joshi et al. (2017), in which they introduced threshold and latency differences between the anodic and cathodic stimulation in order to qualitatively account for the cat ANF data by Miller et al. (1999). However, findings from measurements with human CI users indicate that more information is needed to verify the accurateness such model structures. In contrast to findings from cat ANF recordings (Miller et al., 1999), human CI users tend to be more sensitive to charge-balanced biphasic pulses when the leading excitatory polarity is anodic (Macherey et al., 2008; Undurraga et al., 2010). Further, it has been suggested that the found polarity differences in single-fiber recordings might actually be related to the cochlear location of the stimulating electrode (Ranck, 1975). Another likely explanation for the polarity-dependency of sensitivity around the stimulating electrode is the degree of myelinization and degeneration of peripheral parts of the nuclei near the electrode (McIntyre & Grill, 2000; Rattay et al., 2001; Zhou et al., 1995). Due to the above-mentioned open questions regarding the site of excitation, we decided to use the simpler and common approach in phenomenological models (Takanen et al., 2016) to have the stimulation occur in one distinct location of the auditory nerve fiber in the present model. At its present form, the model does not include differences in absolute threshold values or in latencies for anodic- or cathodic-leading pulses, but such aspects can be explored with the model quite straightforwardly if needed.

Together, the evaluations in the present work and in the earlier work by the group (Horne et al., 2016) have shown that, despite its simplified view of the site of excitation, the model can accurately reproduce physiological data from single pulse stimulations with various (monophasic or symmetric/asymmetric charge-balanced biphasic) pulse shapes. With the latest extensions of the model described in this work, it reproduces also data considering temporal phenomena that affect the responsiveness of the ANF to pulsatile stimulation in modern CIs. Table 2 summarizes the evaluations of the (S-)BLIF model performed in the present study and in Horne et al. (2016). The evaluations of the present study cover only a part of the neurophysiological data available in the literature and several datasets remain to be investigated. Nevertheless, the tested dataset already covers studies from different labs and using various pulse shapes, and the S-BLIF model can reproduce them quantitatively using the same set of parameters, covering phenomena and datasets with which other models have not been evaluated yet.

**Table 2.** Summary of the Evaluations of the (S-)BLIF Model Against Single-Fiber Data from Literature. the Listed Data Cover Simulations Performed by Horne et al. (2016) and the Ones Performed in the Present Work.

Feature	Animal data	Model performance	Note
Spiking probability and its effect on latency	Miller et al. (1999)	Model reproduced the data.	Both data obtained with monophasic pulses.
Dependency of spiking probability on pulse duration	Van Den Honert and Stypulkowski (1984)	Model reproduced the data.	Monophasic pulses used for data collection and simulations.
Effect of IPG on threshold value of symmetric biphasic pulses	Shepherd and Javel (1999)	Model reproduced the data.	
Dependency of threshold value on the pulse shape with pseudo-monophasic pulses	Shepherd, Hardie, and Baxi (2001)	Model reproduced the data.	Charge-balanced triphasic pulses or other pulse shapes not tested.
Refractoriness	Cartee et al. (2000); Dynes (1996); Miller et al. (2001)	Model reproduced the data.	Monophasic pulses used for data collection and simulations.
Facilitation with charge-balanced pulses	Cartee et al. (2000)	Model reproduced the data.	The leaky integration of incoming current simulates facilitation effects also for monophasic pulses, but not accurately enough to reproduce neurophysiological data (Dynes, 1996).
Facilitation and accommodation with pulse trains	Heffer et al. (2010)	Model reproduced the data.	Original animal study likely overestimated the amount of accommodation due to study design.
Accommodation with pulse trains	Miller et al. (2011)	Model reproduced effect of supra-threshold maskers but did not reproduce the effect of high-rate masker at sub-threshold stimulation levels.	A dedicated element for accommodation at sub-threshold levels would be needed to fully reproduce the data.
Spike-rate adaptation effect on spiking efficiency	Javel (1990)	Model reproduced the effect of pulse rate on the spiking efficiency.	Tendency to underestimate the effect for high stimulation rates at above 50% spiking efficiency.
Spike-rate adaptation effect on spike timings	Miller et al. (2008)	Model reproduced the data.	Evaluations limited to measurements within 4–50 ms time frame from the stimulus onset in original data.
Spike-rate adaptation effect on vector-strength values between input and neuron's output.	Dynes and Delgutte (1992); Hartman and Klinke (1990); Miller et al. (2008)	Model reproduced the data.	Data by Dynes and Delgutte (1992) and Hartman and Klinke (1990) collected with sinusoidal electrical stimuli.

The present model offers a versatile instrumental tool for testing new coding strategies employing, e.g., pseudo-monophasic pulses or variable pulse timing. For instance, one can use the model to optimize the timings and amplitudes of the pulses in a stimulation sequence to obtain the desired spiking activity (Seeber & Li, 2022). Due to the limited parameter space, the model has already been shown to be conveniently tunable to individual CI users to predict ECAP responses and hearing percepts (Werner et al., 2018). There, the authors coupled a simple 2-D propagation model with the present S-BLIF model and optimized the neural density and model parameters (average threshold, standard deviation of threshold values among modeled neurons, refractory parameters, and facilitation constant) to successfully predict the loudness growth and ECAP data of individual CI users using the data from Cohen's paper series (2009a, 2009b, 2009c, 2009d, 2009e). Another idea for future work is to extend the S-BLIF model with models of binaural-cue decoding in the superior olivary complex (Grothe et al., 2010; Takanen et al., 2014) to predict CI users’ sensitivity to differences in binaural cues. Pursuing the modeling of hearing outcomes of CI users considering both unilateral and bilateral stimulation provides also clear plan for future work.

## Conclusions

In this study, we present a phenomenological model for electrically stimulated auditory nerve fibers (ANFs) based on single-fiber cat ANF data from literature. The sequential biphasic leaky integrate-and-fire (S-BLIF) model extends the BLIF model (Horne et al., 2016) for pulse-train stimulation by incorporating elements that enable the model to account for the temporal phenomena related to inter-pulse interactions. The extended model:

Reproduces refractoriness data collected with monophasic pulses (Cartee et al., 2000; Dynes, 1996; Miller et al., 2001);

Reproduces facilitation for pseudo-monophasic pulses (Cartee et al., 2000) and facilitation / accommodation in responses to pulse train sequences of symmetric charge-balanced pulses (Heffer et al., 2010);

Reproduces effects of spike-rate adaptation on the neuron's spiking efficiency (Javel, 1990), spike timings (Miller et al., 2008) and vector-strength values between the neuron's spiking activity and pulse train input sequence (Dynes & Delgutte, 1992; Hartman & Klinke, 1990; Miller et al., 2008) for symmetric charge-balanced pulses.

The S-BLIF model is freely available at DOI:10.5281/zenodo.4674563.
